# Improving the accuracy of genomic prediction in dairy cattle using the biologically annotated neural networks framework

**DOI:** 10.1186/s40104-024-01044-1

**Published:** 2024-07-01

**Authors:** Xue Wang, Shaolei Shi, Md. Yousuf Ali Khan, Zhe Zhang, Yi Zhang

**Affiliations:** 1 State Key Laboratory of Animal Biotech Breeding, National Engineering Laboratory for Animal Breeding, Key Laboratory of Animal Genetics, Breeding and Reproduction of Ministry of Agriculture and Rural Affairs, College of Animal Science and Technology, China Agricultural University, Beijing 100193, China; 2 Bangladesh Livestock Research Institute, Dhaka 1341, Bangladesh; 3 Guangdong Laboratory of Lingnan Modern Agriculture, National Engineering Research Center for Breeding Swine Industry, Guangdong Provincial Key Lab of Agro-Animal Genomics and Molecular Breeding, College of Animal Science, South China Agricultural University, Guangzhou 510642, China

**Keywords:** Biologically annotated neural networks, Dairy cattle, Genomic prediction

## Abstract

**Background:**

Biologically annotated neural networks (BANNs) are feedforward Bayesian neural network models that utilize partially connected architectures based on SNP-set annotations. As an interpretable neural network, BANNs model SNP and SNP-set effects in their input and hidden layers, respectively. Furthermore, the weights and connections of the network are regarded as random variables with prior distributions reflecting the manifestation of genetic effects at various genomic scales. However, its application in genomic prediction has yet to be explored.

**Results:**

This study extended the BANNs framework to the area of genomic selection and explored the optimal SNP-set partitioning strategies by using dairy cattle datasets. The SNP-sets were partitioned based on two strategies–gene annotations and 100 kb windows, denoted as BANN_gene and BANN_100kb, respectively. The BANNs model was compared with GBLUP, random forest (RF), BayesB and BayesCπ through five replicates of five-fold cross-validation using genotypic and phenotypic data on milk production traits, type traits, and one health trait of 6,558, 6,210 and 5,962 Chinese Holsteins, respectively. Results showed that the BANNs framework achieves higher genomic prediction accuracy compared to GBLUP, RF and Bayesian methods. Specifically, the BANN_100kb demonstrated superior accuracy and the BANN_gene exhibited generally suboptimal accuracy compared to GBLUP, RF, BayesB and BayesCπ across all traits. The average accuracy improvements of BANN_100kb over GBLUP, RF, BayesB and BayesCπ were 4.86%, 3.95%, 3.84% and 1.92%, and the accuracy of BANN_gene was improved by 3.75%, 2.86%, 2.73% and 0.85% compared to GBLUP, RF, BayesB and BayesCπ, respectively across all seven traits. Meanwhile, both BANN_100kb and BANN_gene yielded lower overall mean square error values than GBLUP, RF and Bayesian methods.

**Conclusion:**

Our findings demonstrated that the BANNs framework performed better than traditional genomic prediction methods in our tested scenarios, and might serve as a promising alternative approach for genomic prediction in dairy cattle.

**Supplementary Information:**

The online version contains supplementary material available at 10.1186/s40104-024-01044-1.

## Background

Genomic selection [1] has significantly shortened the generation interval and increased the annual genetic gain of economic traits in dairy cattle [2–4] with breeding costs reduced by 92% compared to traditional progeny testing [5]. Statistical models serve as one of the key factors affecting the accuracy of genomic selection, consequently exerting an impact on genetic progress. Currently, the most commonly used models for genomic prediction in dairy cattle include the best linear unbiased prediction (BLUP) models that incorporate genomic information [e.g., the genomic BLUP (GBLUP) and single-step GBLUP (ssGBLUP) methods], executed through solving the mixed model equations (MME), as well as the Bayesian methods with various priors that use Markov chain Monte Carlo (MCMC) to estimate the required genetic parameters. However, the utilization of these linear models is often limited by their assumption that genetic variants influence phenotypes only in an additive manner and fail to capture interactions. The exponential growth of large-scale genomic databases provides a unique opportunity to move beyond traditional linear regression frameworks.

Machine learning (ML) algorithms can build complex nonlinear models and allow interaction between features (i.e., markers). Therefore, ML has been considered an effective tool for interpretating massive genomic datasets [6]. Recently, several studies showed that nonlinear ML algorithms typically exhibited higher predictive accuracy than conventional methods such as GBLUP and Bayesian approaches [6–9], especially for complex traits with broad-sense heritability driven by non-additive genetic variation (e.g., gene-gene interactions) [10]. In dairy science, ML has been successfully applied to predict a whole range of different traits, such as milk production [11, 12], mastitis [13], and methane production [14]. Ensemble methods are a category of advanced ML algorithms. Random forest (RF), as an ensemble method, is model specification free and may account for non-additive effects [15]. Moreover, it remains a relatively fast algorithm in ensemble methods even when dealing with a large number of covariates and interactions, making it suitable for both classification and regression problems [15]. Therefore, RF has been widely employed in genomic prediction [9, 15, 16]. Furthermore, to comprehensively capture interactions between markers and non-additive effects, an increasing body of research is being devoted to neural networks [17–19], which reflect the nonlinear relationships between variables by exploiting nonlinear activation functions between network layers. However, conventional neural networks often do not consider the varying influences of different genomic regions on traits, and thus lack certain biological interpretability. Studies have shown that genetic variants do not contribute equally to genetic variance, and genetic variations of large effect on a trait are often distributed within specific genomic regions [20–22]. Based on this framework, new prediction methods have been developed, including BayesRC [23], BayesRS [24], BayesRR-RC [25], NN-Bayes and NN-MM [26].

Most recently, Demetci et al. [27] developed the biologically annotated neural networks (BANNs), a nonlinear probabilistic framework for association mapping in genome-wide association studies (GWAS). BANNs are a class of feedforward Bayesian models that integrate predefined SNP-set annotations, and the BANNs framework has achieved better performance than state-of-the-art methods in the area of GWAS by using prior defined biology information [27]. BANNs employ variational inference for parameter estimation, which is an optimization method that can leverage modern optimization techniques such as Stochastic Gradient Descent (SGD), to find an approximation to the posterior distribution. Consequently, variational inference is often more efficient than MCMC sampling, as the latter requires extensive sampling to estimate the full posterior distribution [28]. Philosophically, compared to traditional linear models, the BANNs framework considers the heterogeneity of the function of SNP-sets according to annotations. BANNs take into account the interactions between markers through setting of neural network layers, which seems theoretically more in line with the biological process of complex traits. However, the existing BANNs framework has not been applied to genomic prediction.

The objectives of this study were to: (i) extend the BANNs framework to the field of dairy cattle genomic selection by exploring the optimal SNP-set partitioning strategies; and (ii) assess the predictive ability of the BANNs framework by comparing it with GBLUP, RF and Bayesian methods.

## Materials and methods

### Statistical models

#### BANNs

As an interpretable neural network, the BANNs framework models SNP effects in the input layers and SNP-set effects in the hidden layers separately. BANNs utilized sparse prior distributions to select variables for network weights. The weights and connections of the network are treated as random variables that present genetic effects at various genomic scales. Moreover, BANNs fall into the category of Bayesian Network (BN) models. BN models can be viewed as a non-conjugate form of Bayesian linear regression, because they automatically learn hyperparameters for priors from the data, making them generally more flexible and better suitable for capturing complex data structures [29].

The model representation for the BANNs framework is as follows:

1 $${\varvec y}=\sum _{g=1}^{G}h\left({\varvec{X}}_{g}{\varvec{\theta }}_{g}+{\boldsymbol 1}{b}_{g}^{\left(1\right)}\right){w}_{g}+{\boldsymbol 1}{b}^{\left(2\right)},$$

where $\varvec{y}$ is the vector of the response variable, that is, standardized de-regressed proofs (DRPs); ${\varvec{X}}_{g}=\left[{\varvec{x}}_{1},\dots ,{\varvec{x}}_{\left|{S}_{g}\right|}\right]$ is a subset of SNPs for SNP-set $g$; ${\varvec{\theta }}_{g}=\left({\theta }_{1},\dots ,{\theta }_{\left|{S}_{g}\right|}\right)$ are the corresponding inner layer weights; $h\left(\bullet \right)$ denotes the nonlinear activations defined for the neurons in the hidden layer; ${\varvec w}=\left({w}_{1},\cdots ,{w}_{G}\right)$ are the weights of the G-predefined SNP-sets in the hidden layer; ${\varvec{b}}^{\left(1\right)}=\left({b}_{1}^{\left(1\right)},\cdots ,{b}_{G}^{\left(1\right)}\right)$ and ${b}^{\left(2\right)}$ are deterministic biases generated during the training phase of the network in the input and hidden layers, respectively; **1** is an N-dimensional vector of ones. For convenience, the genotype matrix (column-wise) and the trait of interest are assumed to be mean-centered and standardized. In this study, $h\left(\bullet \right)$ is defined as the Leaky rectified linear unit (Leaky ReLU) activation function. If *x* > 0, then $h\left(x\right)=x$, otherwise, we define $h\left(x\right)=0.01x$.

The weights of the input layer ($\varvec{\theta }$) and the hidden layer (${\varvec w}$) were treated as random variables, allowing simultaneous multi-scale genomic inference on both SNPs and SNP-sets. SNP-level effects are assumed to follow a sparse K-mixed normal distribution:

2 $${\theta }_{j} \sim {\pi }_{\theta }\sum _{k=1}^{K}{\eta }_{\theta k}N\left(0,{\sigma }_{\theta k}^{2}\right)+\left(1-{\pi }_{\theta }\right){\delta }_{0},$$

where ${\pi }_{\theta }$ represents the total proportion of SNPs that have a non-zero effect on the trait; ${\varvec{\eta }}_{\theta }=\left({\eta }_{\theta 1},\dots ,{\eta }_{\theta k}\right)$ denotes the marginal (unconditional) probability that a randomly selected SNP belongs to the k-th mixture component and that ${\sum }_{k}{\eta }_{\theta \kappa }$=1; ${\varvec{\sigma }}_{\theta }^{2}=\left({\sigma }_{\theta 1}^{2},\dots ,{\sigma }_{\theta K}^{2}\right)$ are the variance of the K non-zero mixture components; and ${\delta }_{0}$ is a point mass at the zero point. The present study follows previous studies and lets K = 3, indicating that SNPs may have large, moderate and small non-zero effects on phenotypic variation [30–32]. To infer the hidden layer, it was assumed that the enriched SNP-sets contain at least one non-zero effect SNP by placing a spike and slab prior to the hidden weights:

3 $${w}_{g} \sim {\pi }_{w}N\left(0,{\sigma }_{w}^{2}\right)+\left(1-{\pi }_{w}\right){\delta }_{0}.$$

Due to the lack of prior knowledge regarding the proportion of relevant SNPs and SNP-sets with non-zero weights, an assumption was made on relatively uniform priors on $\text{log}\left({\pi }_{\theta }\right)$ and $\text{log}\left({\pi }_{w}\right)$ [27]:

4 $$\text{log}\left({\pi }_{\theta }\right)\sim U\left(-\text{log}\left(J\right),\text{log}\left(1\right)\right), \text{log}\left({\pi }_{w}\right)\sim U\left(-\text{log}\left(G\right),\text{log}\left(1\right)\right),$$

where ${\pi }_{\theta }$ denotes the total proportion of SNPs with a non-zero effect on the trait of interest, $J$ denotes the number of SNPs, and ${\pi }_{w}$ denotes the total proportion of annotated SNP-sets enriched for the trait of interest. In addition, the variational Bayesian algorithm was used to estimate all model parameters. In the BANNs framework, the posterior inclusion probabilities (PIPs) provide statistical evidence for the importance of each variant in explaining the overall genetic architecture of a trait. These quantities are defined as the posterior probability that the weight of a given connection in the neural network is non-zero:

5 $$\text{P}\text{I}\text{P}\left(j\right)\equiv \text{P}\text{r}[{\theta }_{j}\ne 0|\varvec{y},\varvec{X}], \text{P}\text{I}\text{P}\left(g\right)\equiv \text{Pr}\left[{w}_{g}\ne 0|\varvec{y},\varvec{X},{\varvec{\theta }}_{g}\right],$$

where $j$ and $g$ represent a specific SNP and a specific SNP-set, respectively.

In addition, the variational expectation-maximization (EM) algorithm was utilized for estimating the parameters of the neural network, and parameters in the variational EM algorithm were initialized through random draws from their assumed prior distributions. The iteration within the algorithm terminates upon meeting one of the following two stopping criteria: (i) the difference between the lower bounds of two consecutive updates falls within the range of 1 × 10^−4^, or (ii) the maximum iteration count of 10,000 is reached [27]. In addition, the initial values of variance ${\sigma }_{0}^{2}$ and the number of models L were set to 0.01 and 20, respectively. In summary, the Bayesian formulation in the BANNs framework makes network sparsity a goal for genomic selection applications through the context-driven sparse shrinkage prior distribution in Eqs. (1–4).

The original BANNs model partitioned SNP-sets according to gene-annotated SNPs. Two strategies were considered in this study to group the SNPs into different sets. Firstly, biological annotations were considered (denoted as BANN_gene). The cattle genome annotation file was obtained from the NCBI website (https://ftp.ensembl.org/pub/release-94/gtf/bos_taurus/) for mapping SNPs to their nearest neighboring genes and aptly annotating them with relevant gene information. Unannotated SNPs located within the same genomic region were denoted as “intergenic regions” between two genes. A total of G = 16,857 SNP-sets were analyzed, consisting of 9,369 intergenic SNP-sets and 7,488 annotated genes. Secondly, 100 kb windows were used to divide SNPs on each chromosome into different groups (denoted as BANN_100kb). A total of G = 22,626 SNP-sets were analyzed using this strategy. On note, the choice of a 100 kb window was based on our testing of the predictive ability with different SNP division intervals (50 kb, 100 kb, 200 kb, 300 kb, 400 kb, 600 kb, 800 kb, 1,000 kb), where we found that dividing based on a 100 kb window yielded better results (results not shown).

#### GBLUP

The model of the GBLUP is given as:

6 $${\varvec y}={\boldsymbol 1}\mu +{\varvec Z}{\varvec g}+{\varvec e},$$

in which ${\varvec y}$ is also the vector of standardized DRPs, $\mu$ is the overall mean, ${\boldsymbol 1}$ is a vector of ones, ${\varvec g}$ is the vector of genomic breeding values, ${\varvec e}$ is the vector of random residuals, and ***Z*** is an incidence matrix allocating records to $\varvec g$. The assumptions of random effects were:

7 $${\varvec g}\sim {\textrm{N}(\varvec{0}},\;{\varvec G}\sigma_{\textrm{g}}^2) {\text{and}}\;{\varvec e}\sim \text{N}(\varvec{0},{\varvec D}\sigma_{e}^2),$$

in which ***G*** is the genomic relationship matrix (***G*** matrix), ***D*** is a diagonal matrix with ${d}_{ii}=\frac{1-{r}_{i}^{2}}{{r}_{i}^{2}}$, (${r}_{i}^{2}$ is the reliability of DRP of individual *i*), and ${\sigma }_{{g}}^{2}$and ${\sigma }_{{e}}^{2}$ are the additive genetic variance and the random residual variance, respectively.

In this study, GBLUP was carried out using DMU software [33]. The AI-REML method in the DMUAI procedure was used to estimate the variance components.

#### BayesB

In BayesB, the proportion of markers with no effect is assumed to be $\pi$, and the proportion of markers with an effect is $1-\pi$, and the prior distribution of SNP effect, ${\beta }_{k}$, was assumed to be *t*-distributed. The formula of BayesB can be written as follows:

8 $${\varvec y}={\boldsymbol 1}\mu +{\sum }_{k=1}^{m}{\varvec{x}}_{\varvec{k}}{\beta }_{k}+{\varvec e},$$

where $\varvec y$ represents the vector of standardized DRPs, ${\varvec{x}}_{\varvec{k}}$ is the vector of genotypes for the k^th^ SNP, and ${\beta }_{k}$ is the effect of the k^th^ SNP. The prior distribution of ${\beta }_{k}$ is as follows:

9 $${\beta}_{k}|{S}_{\beta}^{2},v,\pi \sim IID \left\{\begin{array}{c}0\ with\ probability\ \pi \\ t\left(0,{S}_{\beta }^{2},v\right) with\ probability\ 1-\pi ,\end{array}\right.$$

in which $v$ is the degree of freedom, ${S}_{\beta }^{2}$ is the scale parameter. In the present study, for the BayesB method, we set the proportion of no-effect SNPs ($\pi$) to be 0.95.

#### BayesCπ

In BayesCπ, the marker effects are sampled from a mixture of null and normal distributions. The expression for BayesCπ aligns with that of BayesB except for the prior distribution of ${\upbeta }_{k}$, which is as follows:

10 $${\beta }_{k}|\pi , {\sigma }_{\beta}^{2}\sim IID \left\{\begin{array}{c}0\ with\ probability\ \pi \\ N\left(0,{\sigma }_{\beta}^{2}\right) with\ probability\ 1-\pi, \end{array}\right.$$

where ${\sigma }_{\beta}^{2}$ is the variance of SNP effect. Additionally, in BayesCπ, the value of $\pi$ is treated as an unknown with uniform (0,1) prior and is estimated through sampling [34].

For both BayesB and BayesCπ methods, the MCMC chain was run for 50,000 iterations, the first 20,000 iterations were discarded as burn-in, and every 50 samples of the remaining 30,000 iterations were saved to estimate SNP effects and variance components. The analysis was performed using the Julia package JWAS [35].

#### Random forest

Random forest is a ML algorithm that employs voting or averaging the outcomes of multiple decision trees to determine the classification or predicted values of new instances [36]. Essentially, RF is a collection of decision trees, with each tree exhibiting slight differences from the others. RF reduces the risk of overfitting by averaging the predictions of numerous decision trees [7]. The RF regression can be expressed as follows:

11 $${y}=\frac{1}{\it{M}}\sum\limits_{\it{m}=1}^{\it{M}}{t}_{\it{m}}\left({\psi }_{\it{m}}\left({y}:{\varvec X}\right)\right),$$

where ${y}$ represents the predicted value from the RF regression, ${t}_{m}\left({\psi }_{m}\left({y}:{\varvec X}\right)\right)$ represents an individual regression tree, and $M$ represents the number of decision trees in the forest. Predictions were obtained by propagating predictor variables through the flowchart of each tree, with the estimated values at the terminal nodes serving as the predictions. The final predictions for unobserved data were determined by averaging the predictions across all trees in the RF. To optimize the model, a grid search approach was employed to identify the most suitable hyperparameter $M$ and the maximum tree depth, with an inner five-fold cross-validation (CV) being conducted to tune these hyperparameters.

### Datasets

In this study, phenotypic and genomic data were collected from Chinese Holstein cattle. The population and phenotype information are shown in Table 1. The phenotypic data included three milk production traits: milk yield (MY), fat yield (FY) and protein yield (PY); three type traits: conformation (CONF), feet and leg (FL) and mammary system (MS); and one health trait: somatic cell score (SCS). A total of 6,558, 6,210 and 5,962 individuals were genotyped for milk production traits, type traits and SCS, respectively. DRPs derived from the official estimated breeding values (EBV) provided by the Dairy Association of China following the method proposed by Jairath et al. [37] were used as pseudo-phenotypes for genomic predictions. The DRP reliability for each animal was estimated as ${r}_{DRP}^{2}=\frac{{ERC}_{i}}{{ERC}_{i}+\lambda }$, with $\lambda =\frac{1-{h}^{2}}{{h}^{2}}$, in which ${ERC}_{i}$ refers to the effective record contribution and ${h}^{2}$ refers to the heritability of the trait. Note that ${ERC}_{i}=\lambda \times\frac{{REL}_{i}}{1-{REL}_{i}}$, where ${REL}_{i}$ is the reliability of EBV for individual *i*. All individuals were genotyped using the BovineSNP50 chip containing 54,609 SNPs from Illumina (Illumina, San Diego, CA, USA). Missing genotypes were imputed using Beagle 5.4 [38]. After imputation, SNPs with minor allele frequency (MAF) less than 0.01 and significantly deviating from Hardy-Weinberg equilibrium (*P* < 1.0E-6) were removed using PLINK software [39]. After genotype quality control, 45,944 autosomal SNPs remained for further analyses.

**Table 1:** Summary statistics for the Chinese Holstein cattle population, including the number of genotyped individuals and estimated heritability (*h*^2^)

Trait^a^	*h*^2^	Birth year	Number of genotyped animals
CONF	0.215	1999–2020	6,210
FL	0.193	1999–2020	6,210
MS	0.187	1999–2020	6,210
FY	0.327	1999–2021	6,558
MY	0.335	1999–2021	6,558
PY	0.331	1999–2021	6,558
SCS	0.132	2000–2021	5,962

^a^*CONF* Conformation, *FL* Feet and leg, *MS* Mammary system, *FY* Fat yield, *MY* Milk yield, *PY* Protein yield, *SCS* Somatic cell score

### Cross-validation and genomic prediction accuracy

Prediction accuracy, mean square error (MSE) and dispersion were used to assess the prediction performance of different methods. A 5 × 5 CV (five-fold CV repeated five times, totaling 25 tests) process was carried out. The prediction accuracy was assessed with the Pearson correlation coefficient between standardized DRP (sDRP) and predicted values (PV) of the validation population divided by the mean accuracy $\stackrel{-}{r}$ (square root of reliability) of DRP in validation data:

$$accuracy=\frac{cor\left(sDRP, PV\right)}{\stackrel{-}{r}}.$$

Besides, following the study by Legarra and Reverter [40], the slope of the regression of sDRP on PV was calculated to assess the dispersion of the prediction, although some studies used the regression coefficient as a measure of bias and referred to it as unbiasedness [30, 41]. In addition, MSE was also used as a measure for the performance of different methods, which considered both prediction bias and variability. In each prediction scenario, the reference and validation populations for all methods were the same in each replicate of the five-fold CV, and the final results of accuracy, dispersion and MSE are the averages of five repetitions. Furthermore, multiple* t*-tests were conducted based on the outcomes of five replicates, with *P*-values adjusted using the Bonferroni method, to compare the prediction accuracy of different methods.

### Estimating phenotypic variance explained in the BANNs framework

Given that the BANNs framework offers posterior estimates for all weights in neural networks, it also enables the estimation of phenotypic variance explained (PVE). Here, PVE was defined as the total proportion of phenotypic variation explained by sparse genetic effects (both additive and non-additive effects) [42]. Within the BANNs framework, such estimation can be conducted at both the SNP and SNP-set levels as follows [27]:

$$\text{P}\text{V}\text{E}\left(\varvec{\theta }\right)\approx \frac{\text{V}\left[\varvec{X}{\varvec{\beta }}_{\theta }\right]}{\text{V}\left[\varvec{X}{\varvec{\beta }}_{\theta }\right]+{\tau }_{\theta }^{2}}, \text{P}\text{V}\text{E}\left(\varvec{w}\right)\approx \frac{\text{V}\left[\varvec{H}\left({\varvec{\beta }}_{\theta }\right){\varvec{\beta }}_{w}\right]}{\text{V}\left[\varvec{H}\left({\varvec{\beta }}_{\theta }\right){\varvec{\beta }}_{w}\right]+{\tau }_{w}^{2}},$$

where $\text{V}\left(\bullet \right)$ denotes the variance function, ${\varvec{\beta }}_{\theta }$ and ${\varvec{\beta }}_{w}$ represent the vectors of the marginal posterior means for the input and outer layer weights, respectively. $\varvec{H}\left({\varvec{\beta }}_{\theta }\right)=\left[h\left({\varvec{X}}_{1}{\varvec{\beta }}_{\theta 1}+{b}_{1}^{\left(1\right)}\right),\dots , h({\varvec{X}}_{G}{\varvec{\beta }}_{\theta G}+{b}_{G}^{\left(1\right)})\right]$ represents the matrix of deterministic nonlinear neurons in the hidden layer given ${\varvec{\beta }}_{\theta }$. The estimates of variance hyperparameters ${\tau }_{\theta }^{2}$ and ${\tau }_{w}^{2}$ in the variational EM algorithm were used to approximate the residual variance observed during the two-layer training process [27]. In fact, the formula is similar to the traditional form used for estimating PVE, with the distinction that the contribution of non-additive genetic effects is also taken into account through the nonlinear Leaky ReLU activation function $h\left(\bullet \right)$. In other words, the PVE estimated at the SNP level considers only additive effects, while the PVE estimated at the SNP-set level takes into account both additive and non-additive genetic effects.

## Results

### Annotation summary

**Fig. 1:**
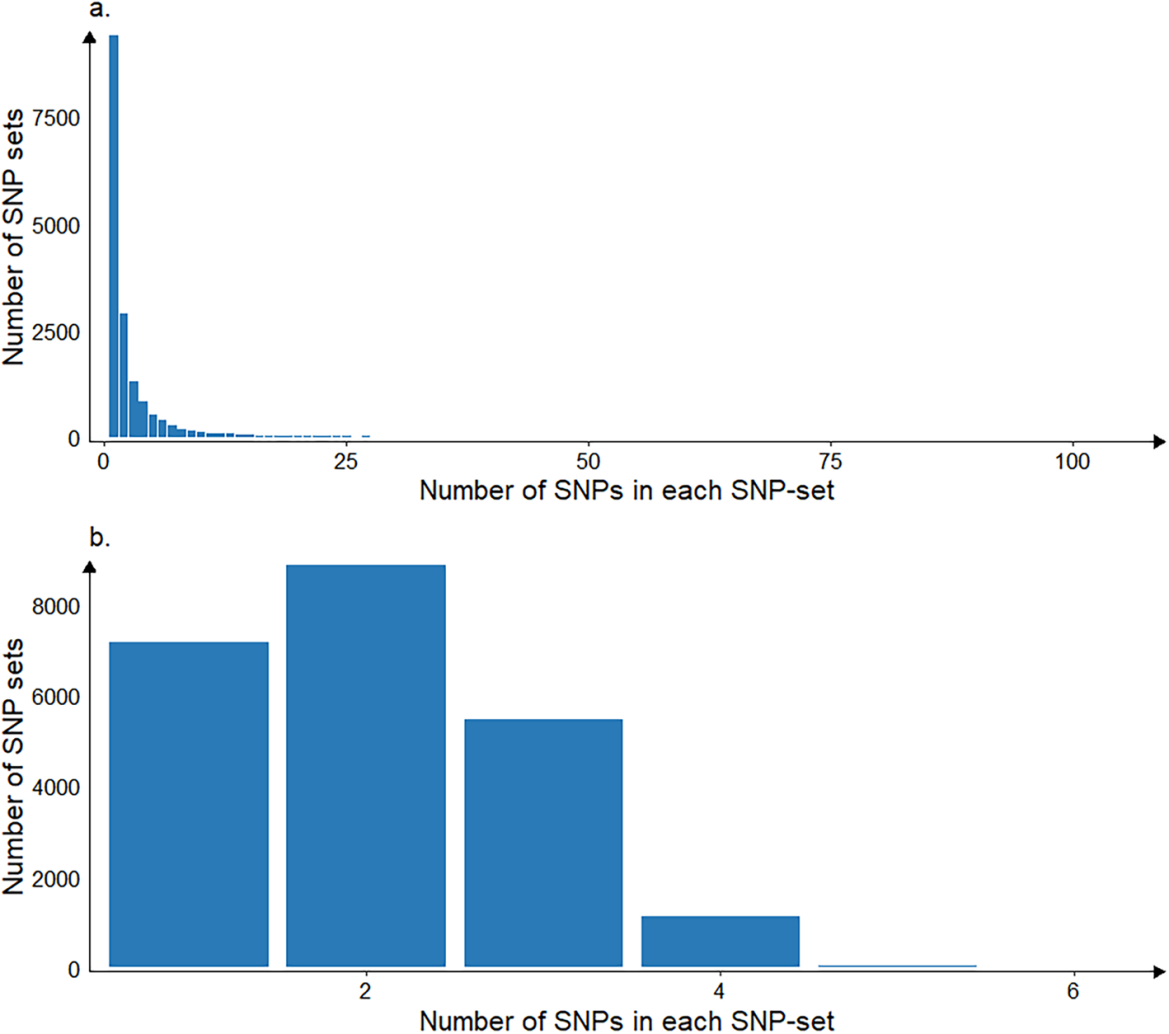
The distribution of the number of SNPs included in each SNP-set under two partitioning schemes. **a** Partitioning SNP-sets according to gene annotation (BANN_gene). **b** Partitioning SNP-sets according to 100 kb physical genomic intervals (BANN_100kb)

The distribution of the number of SNPs in each SNP-set under the two partitioning schemes is shown in Fig. 1. With regards to BANN_gene, of a total of 16,857 SNP-sets, 9,413 contained one SNP (including intergenic regions), while the remaining SNP-sets had varying numbers of SNPs, ranging from 2 to 108. For BANN_100kb, among the 22,626 SNP-sets, 21,466 sets had no more than 3 SNPs (7,152, 8,848 and 5,466 SNP-sets containing 1, 2 and 3 SNPs, respectively), and none of the SNP-sets had more than 6 SNPs. Therefore, it was evident that the distribution of SNPs within BANN_100kb SNP-sets was more uniform than in BANN_gene.

### Genomic prediction accuracy

#### Comparison of prediction performance among BANN_gene, GBLUP, RF and Bayesian methods

**Fig. 2:**
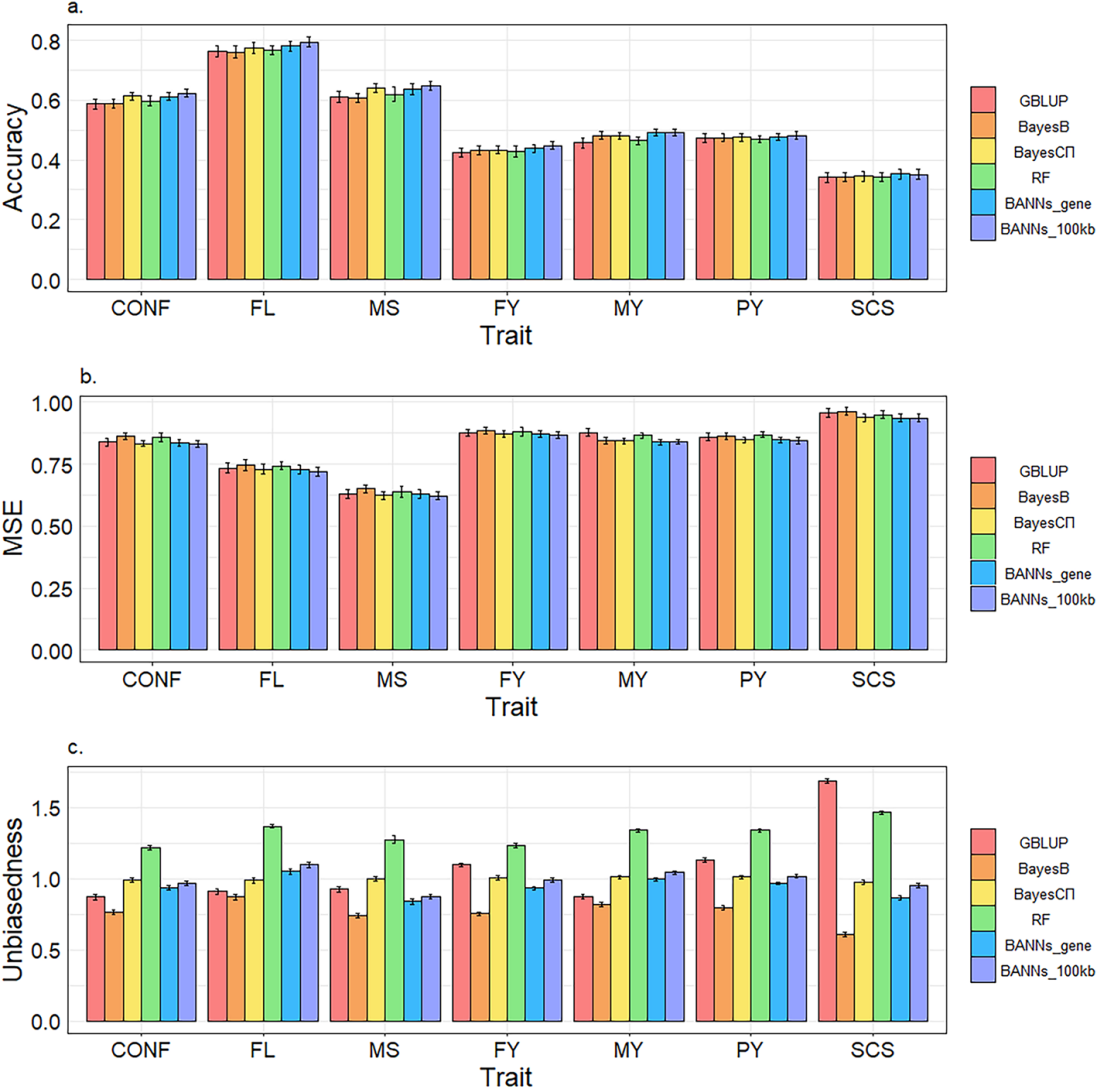
Accuracy (**a**), mean squared error (MSE) (**b**) and dispersion (**c**) of genomic prediction on seven traits of dairy cattle using five-fold cross-validation with five replications. *CONF* Conformation, *FL* Feet and leg, *MS* Mammary system, *FY* Fat yield, *MY* Milk yield, *PY* Protein yield, *SCS* Somatic cell score. The error bar represents the standard error

Figure 2 shows the accuracy, dispersion and MSE of genomic predictions for seven dairy cattle traits using six methods (Table S1 reports the underling values of Fig. 2). In terms of accuracy, BANN_gene performed best compared to GBLUP, RF and Bayesian methods. The average improvement of BANN_gene over GBLUP, RF, BayesB and BayesCπ were 3.75%, 2.86%, 2.73% and 0.85%, respectively, across all seven traits. For milk production traits, BANN_gene demonstrated better performance compared to GBLUP, RF, BayesB or BayesCπ, especially for MY. For example, the accuracy of BANN_gene for MY was 0.491, which resulted in a 7.68% significant improvement compared to GBLUP. The accuracy of BANN_gene for milk production traits, compared to GBLUP, RF, BayesB and BayesCπ, improved by an average of 3.93%, 3.25%, 1.90% and 1.53%, respectively. In case of type traits, BANN_gene significantly outperformed GBLUP, RF and BayesB, while BayesCπ performed similarly with BANN_gene. The improvement of BANN_gene over GBLUP, RF and BayesB was 3.52%, 2.33% and 3.84% on average, respectively.

Compared to GBLUP, RF and Bayesian methods, BANN_gene yielded the lowest or the second lowest MSE. It yielded the smallest MSE for FL, FY, MY and SCS traits, while for other traits, BANN_gene showed the second smallest MSE. However, in terms of overall dispersion, BayesCπ achieved the most appropriate dispersion (i.e., slopes closer to 1), followed by BANN_gene.

In addition, for the comparison of the two Bayesian methods, we found that BayesCπ obtained better results than BayesB across all metrics of accuracy, dispersion, and MSE; besides, as indicated by the estimated standard errors of marker effects (as shown in Table 2), BayesCπ produced smaller standard errors for marker effects across all traits.

**Table 2:** Mean value of the standard error of marker effects estimated by BayesB and BayesCπ methods using all genotyped individuals

Method	Trait^a^						
	CONF	FL	MS	FY	MY	PY	SCS
BayesCπ	2.55 × 10^−6^	4.14 × 10^−6^	2.43 × 10^−6^	3.81 × 10^−6^	3.37 × 10^−6^	3.07 × 10^−6^	2.01 × 10^−6^
BayesB	5.91 × 10^−6^	7.38 × 10^−6^	5.96 × 10^−6^	8.46 × 10^−6^	8.48 × 10^−6^	7.19 × 10^−6^	6.58 × 10^−6^

^a^*CONF* Conformation, *FL* Feet and leg, *MS* Mammary system, *FY* Fat yield, *MY* Milk yield, *PY* Protein yield, *SCS* Somatic cell score

#### Comparison of prediction performance among BANN_100kb, GBLUP, RF and Bayesian methods

BANN_100kb achieved the highest accuracy in all scenarios when compared to the conventional GBLUP and Bayesian methods, where the accuracy of BANN_100kb was improved by an average of 4.86%, 3.95%, 3.84% and 1.92% compared to GBLUP, RF, BayesB and BayesCπ, ranging from 2.12% to 7.46%, 2.63% to 5.38%, 1.87% to 6.93% and 1.25% to 3.23%, respectively. For milk production traits, BANN_100kb consistently achieved the highest accuracy, particularly for FY and MY traits, where BANN_100kb exhibited significant improvements of 5.42% and 7.46%, respectively, compared to GBLUP. Compared to GBLUP, BayesB and BayesCπ, BANN_100kb displayed average improvements in accuracy of 4.48%, 2.45% and 2.08%, respectively. For type traits, BANN_100kb also obtained the highest accuracy, with average improvements over GBLUP, RF, BayesB and BayesCπ of 5.36%, 4.14%, 5.68% and 1.71%, respectively. These results suggest that BANN_100kb captured some intrinsic nonlinear features within the dairy cattle data, whereas GBLUP and Bayesian methods did not. Regarding MSE, BANN_100kb showed the lowest value for all traits. As for dispersion, the dispersions of the four methods were roughly as follows: b_BayesCπ_ < b_BANN_100kb_ < b_GBLUP_ < b_BayesB_ < b_RF_.

#### Comparison of prediction performance between BANN_gene and BANN_100kb

Comparison of the BANNs methods used for differently partitioned SNP subsets (BANN_gene vs. BANN_100kb) showed that BANN_100kb consistently demonstrated superior accuracy with an average improvement of 1.80%, 1.79%, 1.73% and 1.82% over BANN_gene for CONF, FL, MS and FY traits, respectively. However, for MY and SCS traits, the accuracy of BANN_100kb closely resembled that of BANN_gene, with accuracies of 0.49 and 0.491 for MY and 0.351 and 0.352 for SCS. Overall, BANN_100kb resulted in an average improvement of 1.07% compared to BANN_gene across all traits (1.77% for type traits; 0.54% for milk production traits), although the improvements were not significant for most traits.

Concerning MSE, BANN_100kb consistently produced lower MSE than BANN_gene in almost all scenarios. Specially, BANN_100kb had an average MSE that was 0.007 lower than that of BANN_gene for milk production traits and an average MSE that was lower than BANN_gene by 0.0013 for type traits. In terms of dispersion, BANN_100kb achieved a generally more appropriate dispersion compared to BANN_gene for both milk production and type traits.

### Posterior inclusion probabilities in the BANNs framework

Table 3 summarizes the average, maximum and minimum values of PIPs across all variants on SNPs and SNP-sets from the BANNs framework. Since BANN_gene and BANN_100kb shared the same SNP layer, both methods yielded identical PIP results at the SNP level. However, at the SNP-set level, BANN_100kb obtained a lower standard error in PIP across all seven traits compared to BANN_gene, as evidenced by the smaller range between the maximum and minimum PIP values obtained by BANN_100kb. In addition, for both BANN_gene and BANN_100kb methods, the maximum PIP values obtained at the SNP-set level were significantly higher than those at the SNP level for all traits.

**Table 3:** Summary of posterior inclusion probabilities (PIPs) across all variants on SNPs and SNP-sets from the BANNs framework in five replicates of five-fold cross-validation

Trait^a^	Layer	BANN_gene				BANN_100kb			
		Mean	SE	Maximum	Minimum	Mean	SE	Maximum	Minimum
CONF	SNPs	0.091	3.52 × 10^−6^	0.312	0.085	0.091	3.52 × 10^−6^	0.312	0.085
	SNPs-Sets	0.090	4.06 × 10^−5^	0.764	0.069	0.090	2.60 × 10^−5^	0.621	0.073
FL	SNPs	0.091	4.83 × 10^−6^	0.250	0.083	0.091	4.83 × 10^−6^	0.250	0.083
	SNPs-Sets	0.090	5.55 × 10^−5^	0.996	0.064	0.091	3.53 × 10^−5^	0.901	0.069
MS	SNPs	0.091	3.52 × 10^−6^	0.209	0.085	0.091	3.52 × 10^−6^	0.209	0.085
	SNPs-Sets	0.090	4.32 × 10^−5^	0.931	0.068	0.090	2.69 × 10^−5^	0.660	0.072
FY	SNPs	0.091	1.98 × 10^−6^	0.225	0.088	0.091	1.98 × 10^−6^	0.225	0.088
	SNPs-Sets	0.090	4.93 × 10^−5^	0.998	0.066	0.090	3.21 × 10^−5^	0.996	0.071
MY	SNPs	0.091	2.03 × 10^−6^	0.161	0.087	0.091	2.03 × 10^−6^	0.161	0.087
	SNPs-Sets	0.090	5.21 × 10^−5^	1.000	0.065	0.090	3.28 × 10^−5^	0.990	0.070
PY	SNPs	0.091	2.69 × 10^−6^	0.131	0.086	0.091	2.69 × 10^−6^	0.131	0.086
	SNPs-Sets	0.090	5.39 × 10^−5^	1.000	0.066	0.091	3.35 × 10^−5^	0.864	0.071
SCS	SNPs	0.093	2.18 × 10^−5^	0.475	0.069	0.093	2.18 × 10^−5^	0.475	0.069
	SNPs-Sets	0.090	6.02 × 10^−5^	1.000	0.071	0.091	3.51 × 10^−5^	0.837	0.076

^a^*CONF* Conformation, *FL* Feet and leg, *MS* Mammary system, *FY* Fat yield, *MY* Milk yield, *PY* Protein yield, *SCS* Somatic cell score, *SE* standard error

### Estimating phenotypic variance explained in the BANNs framework

**Fig. 3:**
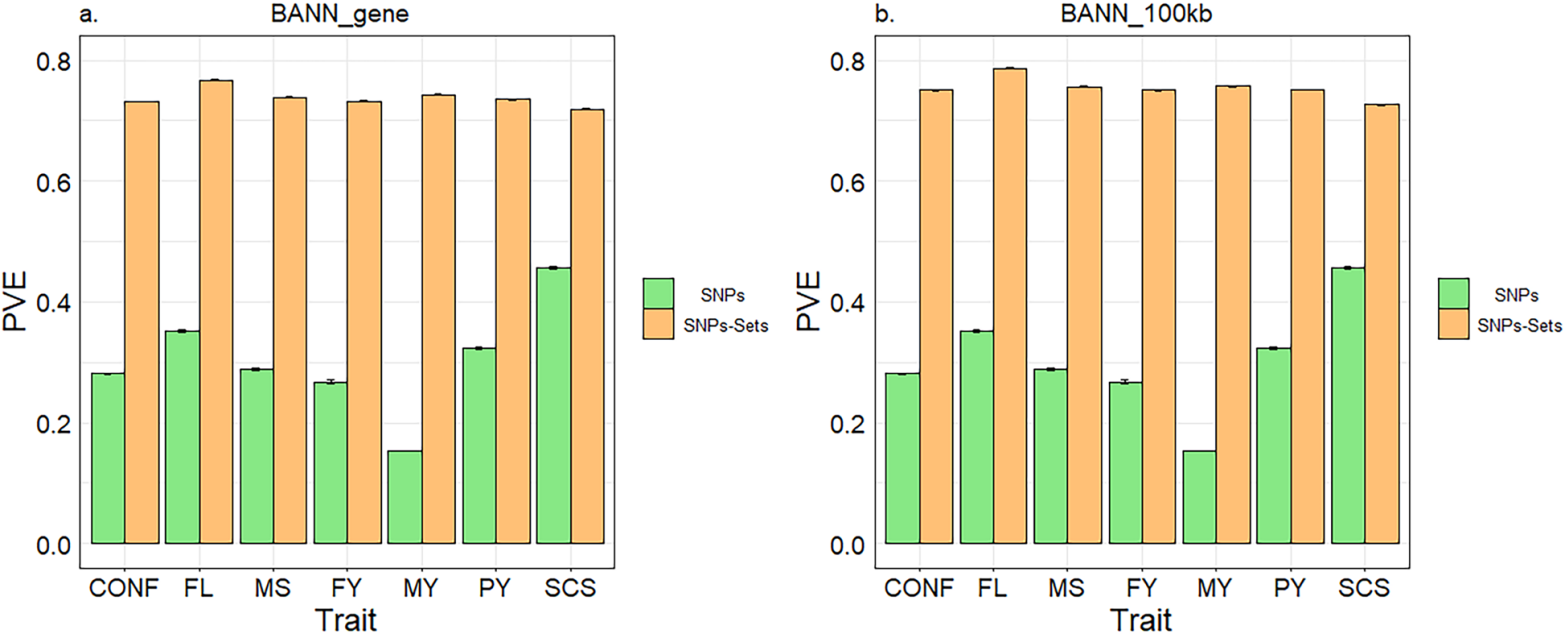
Phenotypic variation explained (PVE) for the seven traits as assessed with five replicates of five-fold CV. **a** PVE estimated using the BANNs_gene method. **b** PVE estimated using the BANNs_100kb method. The error bar represents the standard error

Figure 3 presents the average PVE for the seven traits in five replicates of five-fold CV. For all traits, the PVE estimates obtained at the SNP-set level were substantially greater than those at the SNP level, regardless of whether they were derived from BANN_gene or BANN_100kb. In addition, as BANN_gene and BANN_100kb shared the same SNP layer, they yielded identical PVE estimates at the SNP level, while at the SNP-set level, BANN_100kb obtained larger PVE estimates. The average PVE estimated at the SNP level for both BANN_gene and BANN_100kb was 0.303, while the average PVE estimated at the SNP-set level was 0.738 and 0.754 respectively. Moreover, we observed that at the SNP-set level, the PVE for type traits (i.e., CONF, FL and MS) was generally greater than that for milk production traits (i.e., MY, FY, PY and SCS). For example, BANN_gene and BANN_100kb had average PVEs of 0.732 and 0.746 respectively for milk production traits, while for type traits, their average PVEs were 0.746 and 0.764, respectively. This might partly explain why type traits achieved higher accuracy compared to milk production traits.

### Computation time

The average computation time to complete each fold of five-fold CV for all genomic prediction methods is shown in Table S2. The running time of the methods was measured in minutes on an HP server (CentOS Linux 7.9.2009, 2.5 GHz Intel Xeon processor and 515 GB total memory). Among all methods, GBLUP was the fastest algorithm across all traits, with each fold of CV taking an average of 41.76 min to complete the analysis. The computational efficiency of BayesB, with an average of 132.08 min, was comparable to that of BayesCπ, which averaged 148.91 min. As the BANNs framework involves the construction of neural networks, the computation time for BANN_gene (average 275.79 min) and BANN_100kb (average 284.49 min) was longer, roughly twice that of BayesB or BayesCπ. Additionally, we found that the computational efficiency of RF (average 274.10 min) to be close to that of BANN_gene and BANN_100kb. This may be due to RF being an ensemble algorithm, involving the construction of several hundred decision trees, along with data sampling and feature selection for each tree, leading to its computationally intensive process.

## Discussion

The BANNs framework was extended and applied to genomic prediction of dairy cattle for the first time in this study. In addition, two SNP-set partitioning strategies (based on gene annotations and 100 kb windows) under the BANNs framework were also explored. The superiority of the BANNs methodology was demonstrated by using dairy cattle datasets and comparing them to GBLUP, RF and Bayesian methods (BayesB, BayesCπ). BANN_100kb, which partitioned SNP-sets based on 100 kb intervals, outperformed GBLUP, RF, BayesB and BayesCπ methods in terms of prediction accuracy and MSE across all investigated scenarios.

Non-additive effects often play an important role in the phenotypic variation of complex traits [43]. This is also evident from the PVE results in this study, where the PVE at the SNP-set level, considering both additive and non-additive genetic effects, was substantially higher than the PVE at the SNP level, which accounts only for additive effects (Fig. 3). By incorporating nonlinear Leaky ReLU activation functions within the hidden layer, BANN_100kb effectively captured interactions among input variables, enabling the BANNs framework to model sparse genetic effects that encompass both additive and non-additive effects. In contrast, GBLUP and Bayesian methods focus on additive genetics, overlooking potential complex nonlinear relationships between markers and phenotypes (e.g., dominance, epistasis, genotype by environment interactions) [9]. Additionally, in the BANNs approach, the bias term ${b}_{g}^{\left(1\right)}$ for SNP-sets enables each node in the hidden layer to alter the slope for different genotypic combinations, offering a more flexible estimation of generalized heritability. Theoretically, as more nodes and hidden layers are added to the network architecture, BANNs models will possess an increased capacity to account for non-additive genetic effects, akin to classical Gaussian process regression methods [27]. Consequently, BANNs may exhibit greater advantages when applied to high density SNP markers or whole-genome sequencing (WGS) data, as the use of WGS data has not improved the accuracy of genomic prediction compared to using high-density SNP panels [44, 45]. The BANNs framework could potentially provide a promising direction in this context. This is worth investigating in further studies.

It was found that Bayesian methods generally outperformed GBLUP. Bayesian models’ prediction accuracy is affected by the consistency between the underlying assumptions of the model and the true distribution of marker effects. Bayesian models improved prediction accuracy by shrinking the effects of noisy markers to zero. However, the performance of Bayesian methods over GBLUP mainly depends on the presence of QTLs with large effects on the analyzed trait [46]. As milk production traits (e.g., FY, MY, PY and SCS) were characterized by major effect QTLs [47], both BayesB and BayesCπ outperformed the GBLUP method, which assumed all SNP effects follow the same normal distribution. In addition, GWAS on dairy cattle [48] and beef cattle [49] have found that only a few SNPs were significant for type traits, suggesting that most genetic variants have similar medium or small effects on the traits. This might be the reason for the similar performance of BayesB and GBLUP in type traits (e.g., CONF, FL and MS). Additionally, it was observed that BayesB yielded more over/under dispersion compared to other methods. Despite BayesCπ producing less over/under dispersion, its prediction accuracy and MSE values across all traits still remained inferior to those of BANN_100kb.

In this study, we observed that the predictive performance of BANN_gene was not as strong as that of BANN_100kb. As shown in Fig. 3, the PVE values obtained by BANN_100kb at the SNP-set level were greater than that obtained by BANN_gene at the same level for all traits. This indicates a higher proportion of phenotypic variance explained by genetic effects in the BANN_100kb method, which may partially account for its higher accuracy. In addition, as evidenced by the distribution of SNPs (Fig. 1), the 100 kb interval partitioning method resulted in a more uniform SNP distribution and formed a larger number of SNP-sets (a total of 22,626 SNP-sets). In contrast, with the gene-based partitioning approach, the distribution of SNPs in the SNP-sets was highly uneven (the number of SNPs in each set ranged from 1 to 108) and many SNP-sets contained only one SNP. In fact, BANNs are likely to rank SNP-set enrichments that are driven by just a single SNP as less reliable than enrichments driven by multiple SNPs with nonzero effects [27]. Besides, SNP-sets containing only one SNP struggle to capture interactions or combinatorial effects among multiple loci. When the phenotype is affected by multiple variants within a gene region, a SNP-set containing only one SNP may not represent the total genetic contribution of that region, potentially leading to the model overlooking some biological information and thereby affecting its predictive ability. However, retaining these SNPs might still be beneficial compared to removing them, as BANNs are able to prioritize trait relevant SNPs and SNP-sets [27], and some of these single-SNP sets may contain SNPs that are associated with the traits of interest. In addition, in neural networks, the uneven connectivity between hidden and input layer neurons might also affect the predictive ability of the model, primarily for the following reasons: (I) Uneven connectivity might resulted in an imbalanced weight distribution, causing the network to be unable to capture different aspects of the input data in a balanced manner. This might result in biased feature extraction from the input data, ultimately affecting the model’s generalization ability. (II) Uneven connectivity might lead to unstable gradient updates, resulting in issues such as slow convergence, local optima, gradient explosion, or vanishing gradients during the training process. (III) Due to the uneven connectivity between hidden and input layer neurons, the network might struggle to capture complex relationships and features within the input data. This limitation could have constrained the expressiveness of the network and negatively affected its predictive ability. Consequently, the more uniform distribution of SNPs in BANN_100kb facilitated the network in capturing complex relationships and features within the input data; moreover, the larger number of SNP-sets in BANN_100kb potentially aided the network in extracting more meaningful information. These factors above potentially contributed to the greater advantage of BANN_100kb over BANN_gene. However, when based on high-density SNP panel or WGS data, the number of SNPs within each gene region will significantly increase, enhancing the reliability of SNP-set enrichment rankings [27]. Therefore, BANN_gene may outperform BANN_100kb under these conditions.

Although BANN_100kb has achieved superior predictive performance in this study, there remain several potential extensions to the BANNs framework. (I) It would be beneficial to explore different prior assumptions and consider alternative (more scalable) approaches for approximate Bayesian inference [50]. (II) Employing deep learning techniques by incorporating additional hidden layers in the neural network. (III) Consider environmental covariates (as well as potential genotype by environment interactions) in the model [27]. (IV) Evidence suggested that modeling multiple phenotypes into analytical models often results in a substantial improvement of statistical power [51]; therefore, extending the BANNs framework to accommodate multiple phenotypes and exploiting phenotype correlations to identify pleiotropic epistatic effects might be beneficial. Moreover, investigating the performance of more SNP partitioning strategies through future experiments would be interesting. For example, (i) LD-based partitioning: since the uneven distribution of LD along the genome (i.e., the LD heterogeneity of LD among regions) has a great impact on genomic prediction [52], dividing SNP-sets according to LD structure allows SNPs with higher LD to be grouped together, which may improve the ability to explain genetic variation, thus better reflecting the effects of genomic selection; (ii) function-annotation-based partitioning: the genetic variance explained by different functional regions varies across the entire genome [53, 54], so dividing SNPs based on gene functional regions could make the resulting SNP-sets more biologically meaningful, such as coding region SNPs, non-coding region SNPs, intronic SNPs, etc. Finally, given that BANNs require more computation time compared to conventional methods (as shown in Table S2), further optimization of the BANNs framework code to reduce computation time remains a worthwhile endeavor.

## Conclusions

This study applied the BANNs framework to the field of genomic prediction in dairy cattle, and compared it with GBLUP, RF and Bayesian methods. Our results demonstrated that the BANNs framework holds greater potential for enhancing genomic prediction accuracy than traditional GBLUP, RF and Bayesian methods by modelling interactions between markers, emerging as a novel choice for dairy cattle genomic prediction. Further research might explore the performance of BANNs framework when applied to high density SNP markers and WGS data, together with function-annotation-based partitioning of SNP-sets.

### Supplementary Information

**Additional file 1: Table S1** Accuracy, dispersion, and mean squared error (MSE) of genomic prediction on seven traits of dairy cattle using five-fold cross-validation with five replications; **Table S2** The average computation time to complete each fold of five-fold CV for all genomic prediction methods.

## Data Availability

The datasets used or analyzed during the present study are available from the corresponding author on reasonable request.

## Abbreviations

BANNs: Biologically annotated neural networks
BN: Bayesian network
CONF: Conformation
CV: Cross-validation
DRP: De-regressed proofs
EBV: Estimated breeding values
EM: Expectation-maximization
FL: Feet & leg
FY: Fat yield
GBLUP: Genomic best linear unbiased prediction
GEBVs: Genomic estimated breeding values
G matrix: Genomic relationship matrix
LD: Linkage disequilibrium
MAF: Minor allele frequency
MCMC: Markov chain Monte Carlo
ML: Machine learning
MME: Mixed model equations
MS: Mammary system
MSE: Mean squared error
MY: Milk yield
PIPs: Posterior inclusion probabilities
PV: Predicted values
PVE: Phenotypic variance explained
PY: Protein yield
RF: Random forest
SCS: Somatic cell score
SGD: Stochastic gradient descent
WGS: Whole-genome sequencing
