# Supplementary material for: Improving the accuracy of genomic prediction in dairy cattle using the biologically annotated neural networks framework
Source: J Anim Sci Biotechnol. 2024 Jul 1;15:87. doi: 10.1186/s40104-024-01044-1 (PMC11215832; doi:10.1186/s40104-024-01044-1)
Supplement: Supplementary file 1 — Additional file 1: Table S1 Accuracy, dispersion, and mean squared error (MSE) of genomic prediction on seven traits of dairy cattle using five-fold cross-validation with five replications; Table S2 The average computation time to complete each fold of five-fold CV for all genomic prediction methods. [file 40104_2024_1044_MOESM1_ESM.docx]

**Table S1** Accuracy, dispersion, and mean squared error (MSE) of genomic prediction on seven traits of dairy cattle using five-fold cross-validation with five replications

| **Metrics^1^** | **Trait^2^** | **Method** | | | | | |
| --- | --- | --- | --- | --- | --- | --- | --- |
|  |  | **GBLUP** | **BayesB** | **BayesCπ** | **RF** | **BANN_gene** | **BANN_100kb** |
| Accuracy | CONF | 0.587 ± 0.017^b^ | 0.588 ± 0.014^b^ | 0.614 ± 0.013^a^ | 0.596±0.017^b^ | 0.611 ± 0.013^a^ | **0.622** ± 0.013^a^ |
|  | FL | 0.763 ± 0.02^d^ | 0.761 ± 0.021^d^ | 0.774 ± 0.020^bc^ | 0.768±0.015^cd^ | 0.780 ± 0.018^b^ | **0.794** ± 0.017^a^ |
|  | MS | 0.611 ± 0.018^bc^ | 0.606 ± 0.016^c^ | 0.640 ± 0.016^a^ | 0.619±0.024^b^ | 0.637 ± 0.018^a^ | **0.648** ± 0.016^a^ |
|  | FY | 0.424 ± 0.014^c^ | 0.431 ± 0.014^bc^ | 0.433 ± 0.014^b^ | 0.428±0.018^c^ | 0.439 ± 0.013^ab^ | **0.447** ± 0.013^a^ |
|  | MY | 0.456 ± 0.016^b^ | 0.481 ± 0.013^a^ | 0.480 ± 0.012^a^ | 0.465±0.013^b^ | **0.491** ± 0.010^a^ | 0.490 ± 0.011^a^ |
|  | PY | 0.472 ± 0.016^ab^ | 0.473 ± 0.013^ab^ | 0.476 ± 0.013^ab^ | 0.469±0.013^b^ | 0.478 ± 0.011^ab^ | **0.482** ± 0.012^a^ |
|  | SCS | 0.341 ± 0.018^a^ | 0.343 ± 0.014^a^ | 0.345 ± 0.016^a^ | 0.342±0.015^a^ | **0.352** ± 0.016^a^ | 0.351 ± 0.016^a^ |
| Dispersion | CONF | 0.870 ± 0.036 | 0.762 ± 0.025 | **0.990** ± 0.037 | 1.216±0.064 | 0.936 ± 0.027 | 0.966 ± 0.031 |
|  | FL | 0.910 ± 0.026 | 0.871 ± 0.025 | **0.987** ± 0.028 | 1.369±0.058 | 1.049 ± 0.022 | 1.096 ± 0.021 |
|  | MS | 0.926 ± 0.038 | 0.743 ± 0.025 | **0.998** ± 0.038 | 1.275±0.092 | 0.840 ± 0.030 | 0.870 ± 0.029 |
|  | FY | 1.096 ± 0.047 | 0.752 ± 0.023 | **1.007** ± 0.039 | 1.231±0.079 | 0.934 ± 0.031 | 0.991 ± 0.035 |
|  | MY | 0.871 ± 0.080 | 0.817 ± 0.023 | 1.013 ± 0.033 | 1.338±0.084 | **0.994** ± 0.026 | 1.042 ± 0.026 |
|  | PY | 1.128 ± 0.044 | 0.795 ± 0.022 | **1.012** ± 0.034 | 1.340±0.077 | 0.967 ± 0.024 | 1.017 ± 0.026 |
|  | SCS | 1.685 ± 0.113 | 0.607 ± 0.032 | **0.977** ± 0.063 | 1.463±0.105 | 0.865 ± 0.051 | 0.950 ± 0.055 |
| MSE | CONF | 0.838 ± 0.023 | 0.863 ± 0.022 | 0.833 ± 0.023 | 0.859±0.026 | 0.837 ± 0.024 | **0.831** ± 0.023 |
|  | FL | 0.733 ± 0.016 | 0.746 ± 0.018 | 0.730 ± 0.016 | 0.743±0.015 | 0.729 ± 0.016 | **0.720** ± 0.015 |
|  | MS | 0.629 ± 0.017 | 0.651 ± 0.016 | 0.623 ± 0.016 | 0.638±0.017 | 0.628 ± 0.017 | **0.622** ± 0.016 |
|  | FY | 0.875 ± 0.016 | 0.886 ± 0.016 | 0.873 ± 0.014 | 0.882±0.015 | 0.871 ± 0.013 | **0.866** ± 0.013 |
|  | MY | 0.878 ± 0.023 | 0.844 ± 0.018 | 0.843 ± 0.018 | 0.865±0.019 | **0.838** ± 0.017 | 0.840 ± 0.017 |
|  | PY | 0.860 ± 0.016 | 0.861 ± 0.015 | 0.847 ± 0.014 | 0.869±0.015 | 0.847 ± 0.014 | **0.845** ± 0.014 |
|  | SCS | 0.955 ± 0.02 | 0.963 ± 0.019 | 0.938 ± 0.019 | 0.949±0.018 | **0.936** ± 0.019 | **0.936** ± 0.019 |

^1^ Accuracy: the correlation between standardized DRP and predicted values of the validation population divided by the mean accuracy of DRP in validation data; Dispersion: the slope of the regression of standardized DRP onto the predicted values

^2^ CONF: conformation; FL: feet and leg; MS: mammary system; FY: fat yield; MY: milk yield; PY: protein yield; SCS: somatic cell score

Bold fonts in the same raw denotes the highest prediction accuracy, the lowest bias, or the smallest mean square error (MSE)

^a–d^ Different letters within the same row indicate significant difference as determined by multiple *t*-tests (*P* < 0.05)

**Table S2** The average computation time to complete each fold of five-fold CV for all genomic prediction methods

| **Trait^a^** | **Method** | | | | | |
| --- | --- | --- | --- | --- | --- | --- |
|  | **GBLUP** | **BayesB** | **BayesCπ** | **RF** | **BANN_gene** | **BANN_100kb** |
| CONF | 39.92min | 122.48min | 132.60min | 256.08min | 266.55min | 275.48min |
| FL | 40.48min | 121.53min | 136.38min | 252.62min | 265.52min | 272.53min |
| MS | 39.43min | 122.98min | 131.42min | 253.30min | 269.32min | 276.43min |
| FY | 46.35min | 146.93min | 163.47min | 291.22min | 276.83min | 287.57min |
| MY | 45.22min | 144.17min | 162.18min | 302.23min | 283.27min | 299.13min |
| PY | 45.85min | 151.18min | 178.08min | 296.15min | 297.63min | 303.25min |
| SCS | 35.05min | 115.30min | 138.27min | 267.12min | 271.42min | 277.03min |
| **Average** | **41.76min** | **132.08min** | **148.91min** | **274.10min** | **275.79min** | **284.49min** |

^a^ CONF: conformation; FL: feet & leg; MS: mammary system; FY: fat yield; MY: milk yield; PY: protein yield; SCS: somatic cell score

The running time of the methods was measured on an HP server (CentOS Linux 7.9.2009, 2.5 GHz Intel Xeon processor and 515 GB total memory)
